# FAPs orchestrate homeostasis of muscle physiology and pathophysiology

**DOI:** 10.1096/fj.202400381R

**Published:** 2024-12-16

**Authors:** Kai Yin, Chengmin Zhang, Zihan Deng, Xiaoyu Wei, Tingwen Xiang, Chuan Yang, Can Chen, Yueqi Chen, Fei Luo

**Affiliations:** ^1^ Department of Orthopedics Southwest Hospital, Third Military Medical University (Army Medical University) Chongqing People's Republic of China; ^2^ Department of Biomedical Materials Science Third Military Medical University (Army Medical University) Chongqing People's Republic of China; ^3^ Department for Combat Casualty Care Training Training Base for Army Health Care, Army Medical University (Third Military Medical University) Chongqing People's Republic of China

**Keywords:** fibro/adipogenic progenitors, muscle weakness and fatigue, muscular dystrophy, myopathy muscle disorder

## Abstract

As a common clinical manifestation, muscle weakness is prevalent in people with mobility disorders. Further studies of muscle weakness have found that patients with muscle weakness present with persistent muscle inflammation, loss of muscle fibers, fat infiltration, and interstitial fibrosis. Therefore, we propose the concept of muscle microenvironment homeostasis, which explains the abnormal pathological changes in muscles through the imbalance of muscle microenvironment homeostasis. And we identified an interstitial progenitor cell FAP during the transition from normal muscle microenvironment homeostasis to muscle microenvironment imbalance caused by muscle damage diseases. As a kind of pluripotent stem cell, FAPs do not participate in myogenic differentiation, but can differentiate into fibroblasts, adipocytes, osteoblasts, and chondrocytes. As a kind of mesenchymal progenitor cell, it is involved in the generation of extracellular matrix, regulate muscle regeneration, and maintain neuromuscular junction. However, the muscle microenvironment is disrupted by the causative factors, and the abnormal activities of FAPs eventually contribute to the complex pathological changes in muscles. Targeting the mechanisms of these muscle pathological changes, we have identified appropriate signaling targets for FAPs to improve and even treat muscle damage diseases. In this review, we propose the construction of muscle microenvironmental homeostasis and find the key cells that cause pathological changes in muscle after homeostasis is broken. By studying the mechanism of abnormal differentiation and apoptosis of FAPs, we found a strategy to inhibit the abnormal pathological changes in muscle damage diseases and improve muscle regeneration.

## Graphical abstract

After muscle microenvironment homeostasis is broken, the signal network composed of inflammatory factors, cytokines, and cell membrane ligands was disrupted, resulting in increased transcription of fibrotic genes and decreased inhibition of fat transcription signals in FAP nuclei. Abnormal differentiation of FAP eventually causes pathological changes in muscles.

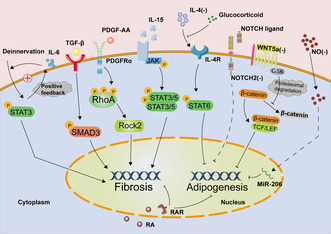

**Abbreviations**
ACE: angiotensin‐converting enzyme
Akt: protein kinase B
ALS: amyotrophic lateral sclerosis
BAT: beige adipose tissue
Bmp3b: bone morphogenetic protein 3B
CTX: cardiotoxin
CXCR4: C‐X‐C chemokine receptor type 4
DMD: Duchenne muscular dystrophy
ECM: extracellular matrix
EGF: epidermal growth factor
EVs: extracellular vesicles
FAPs: fibro/adipogenic progenitors
FDA: Food and Drug Administration
FI: fat infiltration
GDF10: growth differentiation factor 10
GSK3: glycogen synthase kinase 3
HDACi: histone deacetylase inhibitors
HGF: hepatocyte growth factor
IFN‐γ: interferon gamma
IL‐1β: interleukin‐1β
IL‐10: interleukin‐10
IL‐15: interleukin‐15
IL‐33: interleukin‐33
IL‐4: interleukin‐4
IL‐6: interleukin‐6
IMAT: intermuscular adipose tissue
IVDD: intervertebral disk degeneration
Jak–STAT pathway: Janus kinase‐signal transducer and activator of transcription
LBP: low back pain
MCT: muscle connective tissue
MPCs: muscle progenitor/stem cells
MPs: myogenic progenitor cells
MuSCs: muscle satellite cells
NMJ: neuromuscular junction
nNOS: neuronal nitric oxide synthase
PDGF‐AA: platelet‐derived growth factor AA
PDGFR‐α: platelet‐derived growth factor receptor alpha
PPAR‐γ: Ppar‐γ, peroxisome proliferator‐activated receptor gamma
QSC: quiescent satellite cells
RA: retinoic acid
RhoA: Ras homolog gene family, member A
ROCK2: Rho‐associated, coiled‐coil containing protein kinase
RT: resistance training
SASP: senescence associated secretory phenotype factors
SC: satellite cells
SDF‐1: stromal cell‐derived factors
SMA: spinal muscular atrophy
ST2: suppression of tumorigenicity 2 receptor
STAT3: signal transducer and activator of transcription 3
T2DM: diabetes mellitus type 2
THBS1: thrombospondin 1
TK: thymidine kinase
TNF‐α: tumor necrosis factor alpha
Treg cells: regulatory T cells
UCP‐1: uncoupling protein 1
VEGF: vascular endothelial growth factor
WISP1: WNT1‐induced signaling pathway protein 1

## 1 INTRODUCTION

Muscle weakness is a common symptom of muscle degeneration in many types of muscle injury diseases, usually manifested as muscle atrophy and decreased performance. About 5% of American adults aged 60 and older suffer from muscle weakness.^1^ Aging is the main cause of muscle weakness and is diagnosed as sarcopenia when a certain threshold is reached. Moreover, increased infiltration of muscle fat caused by aging, which turns to the muscle strength loss in the other way.^2^, ^3^ The increase in aging population leads to the prevalence of muscle‐damaging diseases, which ultimately intensifies the social medical burden.

In addition to aging, muscle weakness occurs in most muscle‐damaging diseases, such as genetic diseases, mechanical muscle damages, and metabolic disorders, which ultimately lead to physical disability and dysfunction.^1^ Other types of musculoskeletal disorders such as Duchenne muscular dystrophy (DMD) and rotator cuff tear also cause pathological changes associated with muscle weakness. Remarkably, the maintenance of muscle homeostasis is also affected by the metabolic state of the body, so the effect of metabolic diseases on muscle weakness is worth in‐depth study.

The mechanisms that lead to the occurrence of muscle weakness are complex, which are closely relevant to the homeostatic imbalance of muscle microenvironment. FAPs play a crucial role in the maintenance of muscle homeostasis. Interestingly, they are also involved in pathological changes in muscles.

In mouse muscle, the fibro/adipogenic progenitors (FAPs) immunophenotype are Sca‐1 + CD34 + PDGFR‐α+.^4^, ^5^ Besides, the immunophenotype of human muscle mesenchymal progenitor cells is CD15 + CD56 PDGFR‐α+.^6^ More studies have shown that these several immunophenotypes specifically label the same mesenchymal progenitor cells residing in skeletal muscle interstitium.^7^ Therefore, PDGFR‐α is commonly used as a specific molecular marker for screening FAPs in mouse and human tissues. MCT fibroblasts associated with axial muscle may be derived from somatic cells.^8^ And Limb muscle‐associated MCT fibroblasts originate from the lateral plate mesoderm.^9^, ^10^ Finally, MCT fibroblasts in head and neck muscles originate from neural crest cells.^11^, ^12^ As a pluripotent progenitor cell, FAPs able to differentiate into adipocytes, fibroblasts, osteoblasts, and possibly chondrocytes.^4^, ^5^, ^13^ Although FAPs are not involved in myogenic differentiation, they are closely related to the growth and maintenance of resting muscles.^14^, ^15^ FAPs contribute to the normal expansion of the MuSC pool and are indirectly involved in the maintenance of skeletal muscle mass, while ablation of FAPs leads to reduced MuSC expansion and skeletal muscle atrophy.^16^ Notably, FAPs act as a mesenchymal progenitor cell to help them renew and build muscle stem cell niches by producing extracellular matrix (ECM) components.^17^ FAPs communicate closely with other cell types in the muscle microenvironment, which contributes to muscle regeneration and repair after acute injury. For example, the activation of immune cells stimulates the proliferation of FAPs after acute muscle injury, and the activation of FAPs further promotes the proliferation and differentiation of satellite cells (SCs) to regulate muscle regeneration.^18^, ^19^ In addition, soluble molecular‐mediated signaling pathways are also involved in the repair of damaged muscles.^20^, ^21^ After the acute phase of muscle injury, the apoptosis program is initiated to avoid the progression of muscle fat infiltration and fibrosis caused by FAP accumulation.^19^ In summary, FAPs contribute to muscle maintenance and regeneration processes regulated by intracellular/extracellular signaling pathways.

The homeostasis of the muscle microenvironment is disrupted by external stimuli, and the changes in the signaling pathway related to FAPs lead to the occurrence of muscle‐damaging diseases. Examples include sarcopenia, DMD, muscle laceration, intervertebral disk degeneration (IVDD), and T2DM. The occurrence of these muscle‐damaging diseases is mostly related to aging, and motor deficits/immobility disorders contribute to the progression of the disease, ultimately leading to disability of the musculoskeletal system and poor prognosis in elderly patients. Therefore, these muscle‐damaging diseases often have similar pathological manifestations, especially at the histological level of the muscles. Imbalance of muscle microenvironment homeostasis caused by injury or aging leads to weakened regenerative function of muscle stem cell population. Besides, continuous protein breakdown and apoptosis of abnormal cells lead to muscle fiber atrophy. Meanwhile, the function of mesenchymal stem cells is affected by the surrounding environment; muscle repair signals are suppressed, but the tendency of fiber differentiation and fat differentiation increases. Eventually, atrophy of muscle fibers and abnormal changes in muscle interstitium lead to decreased muscle mass and performance, which leading to dyskinesia of body. FAPs play a pivotal role in the deterioration of muscular interstitial. The number of FAPs and the signaling mechanism changes with the progression of aging. During muscle aging, the number of FAPs decreased and their ability to proliferate is weakened, but susceptibility to fibrotic differentiation and fat differentiation increases, which result the increased fibrous tissue and muscle fat infiltration.^5^, ^21^, ^22^ At the same time, the ability of FAPs to maintain normal muscles also declines with age, which is a risk factor for sarcopenia.^23^ FAPs also play a crucial role in inherited skeletal muscle disorders such as DMD. DMD is characterized by the progressive destruction of skeletal muscle, which is subsequently replaced by fat and fibrotic tissues and eventually leading to the early death.^23^ The abnormal behavior of FAPs is strongly associated with the progression of muscular dystrophy in this process. Furthermore, there is a correlation between the behavior of FAPs and endocrine diseases. The literature suggests that patients with T2DM have greater decline in muscle mass, strength, and function with age.^24^ Another common observation is the ectopic lipid deposition between skeletal muscle fibers,^25^, ^26^ which is linked to impaired insulin sensitivity^27^ and decreased muscle function. Interestingly, the deposition of ectopic fat which observed in regenerative muscle of diabetic mice was confirmed to be derived from FAPs.^28^ Therefore, the effect of endocrine diseases on muscle microenvironment is still worthy of further study.

This review discusses the origin and identification of FAPs and their role in skeletal muscle homeostasis. Then, we introduce the occurrence of muscle damage diseases caused by the disruption of muscle microenvironment homeostasis due to external stimuli or aging, as well as similar pathological changes in these diseases. By studying the mechanism of pathological changes in FAPs, we are looking for ways to inhibit abnormal changes in skeletal muscle to prevent and even treat the diseases.

## 2 FAPs PARTICIPATE IN THE REGULATION OF HOMEOSTASIS

### 2.1 The composition of muscle microenvironment

Numerous studies have explored stem cell niches in muscle which establish a stable external environment for stem cells and aid muscle regeneration after injury. However, few papers have discussed the construction of muscle microenvironment and its role in muscle homeostasis. The mechanism of pathological changes after the imbalance of muscle microenvironment homeostasis is also worthy of further study, which could help us find interventions at the cellular level to treat musculoskeletal degenerative diseases. The construction of muscle microenvironment will start from muscle fibers, ECM, intramuscular cell population, vascular cells, neuromuscular junctions (NMJ), and immune cells to study the regulation of intercellular and intermolecular interactions in muscle homeostasis and muscle repair.

Muscle fibers are an important part of the muscle microenvironment, which not only participate in movement but also have certain secretory functions. The cytokines produced and released by muscle fibers are known as myokines. Common myokines such as myostatin, IL‐6, and IL‐7 are involved in muscle hypertrophy and myogenic regulation.^29^ In addition, upregulation of the transmembrane Notch ligand Delta was found in post‐injury muscle fibers, which followed by activation of the Notch signaling cascade in SCs, ultimately inducing proliferation of SCs.^30^ Satellite cell‐derived muscle fibers can produce cytokines that strongly inhibit PDGFR‐α+ cell adipose differentiation.^5^

In addition to muscle fibers, there are large number of ECM components in the muscle microenvironment that are also important in the muscle microenvironment. The ECM network composed of large number of ECM molecules which synthesized and secreted by cells such as fibroblasts and myoblasts, including type IV collagen, laminin, actin, glycoproteins, and other proteoglycans.^31^ The ECM network helps activate SCs and growth factors to maintain normal physiological function of muscles.^32^, ^33^

A variety of cells are present in the muscle microenvironment. Unbiased clustering of the major monocyte types presents in skeletal muscle by using single‐nucleus RNA sequencing revealed all the major cell types expected in skeletal muscle, including SCs, FAPs, endothelial cells, smooth muscle cells, immune cells, and myonuclei.^34^ Satellite cells proliferate in an asymmetrical division manner to maintain stem cell pools and replace damaged myofibers during daily activities. Satellite cells are activated when muscle tissue is damaged; then, new stem cells and many proliferating myoblasts are produced through symmetrical cell division.^35^ In addition to the muscle stem cell population, there are also large number of mesenchymal cells in the muscle interstitium, which are found in the stromal tissue between the basal layer surrounding the skeletal muscle and the outer sheath of the muscle. Fibroblasts are the main components of mesenchymal stromal cell population, and they are one of the architects of skeletal muscle ECM.^36^ There are different subtypes of fibroblasts in different tissue layers, such as perimysial cells, paramysial cells, and endomysial fibroblasts,^37^ which reflect the differences in the production and maintenance of ECM. This suggests that fibroblasts customize ECM production in a location‐specific manner, which plays an important role in the structural integrity and function of skeletal muscle. As a kind of mesenchymal bipotent cell that can differentiate into fibroblasts and adipocytes in vitro, FAPs are also involved in the regulation of muscle microenvironment homeostasis, which will be discussed in detail below.

Endothelial cell is an important part of the muscle microenvironment. Endothelial cells and SCs proliferate synchronously after muscle injury, and neovascularization is accompanied by muscle fiber repair.^38^ Some endothelium‐derived factors, such as vascular endothelial growth factor (VEGF) and hepatocyte growth factor (HGF), can directly affect muscle regeneration.^39^, ^40^ Satellite cells recruit endothelial cells through the secretion of VEGF, and large number of aggregated endothelial cells in turn provide Notch ligand delta‐like protein 4 to activate Notch signaling in SCs, ultimately helping SCs to quiescence and self‐renew.^41^

Innervation is essential for homeostasis of the muscle microenvironment. Plenty of evidence suggests denervation of muscles can lead to progressive atrophy of skeletal muscle. In acute muscle denervation experiments, the number of SC increases during the first week, with a proliferative phase that resembles muscle injury.^42^ However, significant decrease in the number of SCs was detected in long‐term denervated muscles.^43^ This means that long‐term denervation may cause SCs to lose the ability to self‐renew or even enter the mitotic cell cycle.^44^ Secondly, muscles at 6 and 10 weeks of denervation showed a significant increase in SC apoptosis compared with muscles with normal innervation.^45^ On the contrary, SC depletion was found to exacerbate reinnervation and postsynaptic morphological defects in regenerative NMJ, leading to further decline in NMJ and skeletal muscle.^46^

Immune cells are also involved in muscle microenvironment regulation. Only a small number of immune cells are present in normal skeletal muscle, but the number of immune cells in the muscle microenvironment increases rapidly after muscle injury. Neutrophils arrive at the injury site first, then circulate monocytes and macrophages extravasate, and enter the muscle environment rich in pro‐inflammatory cytokines, includes interferon gamma (IFN‐γ) and tumor necrosis factor (TNF).^47^, ^48^ These cytokines can activate macrophages into pro‐inflammatory phenotypes, and these macrophages are named M1 macrophages.^49^, ^50^ Pro‐inflammatory macrophages promote SCs activation and proliferation during the initial muscle regeneration phase.^51^ Subsequently, the phenotypic transformation of pro‐inflammatory macrophages into anti‐inflammatory macrophages stimulates SC differentiation and completes muscle regeneration.^52^ Surely, immune cells cooperate with other intramuscular cell populations to regulate muscle microenvironmental homeostasis. For example, macrophages secrete TNF‐α to interfere with the expansion of the FAP population.^19^ Eosinophil‐derived IL‐4 signaling supports myogenesis by promoting the proliferation of FAPs while limiting their adipogenic differentiation.^18^ Lymphoid cells, especially CD4 regulatory T cells (Treg cells), are also involved in muscle homeostasis regulation. Treg cells directly promote myoblast differentiation through amphiregulin,^53^ an EGF family growth factor, also promote anti‐inflammatory macrophage transformation.^54^ Ablation of Treg cells limits muscle repair^53^ (Table 1).

**TABLE 1:** Composition and function of muscle microenvironment.

Components	Classification	Function	References
Muscle fibers	Type I or type II fibers	Secretion, induce SC proliferation, and inhibit fat differentiation of FAPs.	[5, 29, 30]
Extracellular matrix	Type IV collagen, laminin, glycoproteins, and other proteoglycans	Activate SCs, promote myogenesis and muscle regeneration.	[31, 32, 33]
Intramuscular cell populations	SCs	Myogenesis and muscle maintenance	[34]
	Mesenchymal cells	Generate ECM	[35]
	FAPs	Regulate muscle homeostasis	[14, 17, 18, 19, 20, 21, 22, 55]
Vascular cells	Endothelial cells, pericytes	Neovascularization, muscle regeneration, SC renewal	[36, 37, 38, 39]
Neuromuscular junctions	Presynaptic terminal, synaptic clefts, and postsynaptic membrane	Maintain SC number and muscle mass	[41, 42, 43]
Immune cells	Neutrophils	Recruit macrophages	[45, 46]
	Macrophages	Promote satellite cell proliferation and differentiation and regulate the number of FAPs	[19, 49, 50]
	Eosinophils	Promote FAP proliferation and inhibit FAP adipocyte differentiation	[18]
	Treg cells	Promote myoblast differentiation	[51, 52]

Abbreviations: ECM, extracellular matrix; FAP, fibro/adipogenic progenitors; SC, satellite cells.

### 2.2 FAP in muscle microenvironment homeostasis

As mesenchymal progenitor cells, the role of FAP is especially important for maintenance of muscle microenvironment homeostasis. On the one hand, FAP is defined as a pluripotent progenitor cell, although FAP is not involved in myogenic differentiation, having the ability to differentiate into fibroblasts,^56^ adipocytes,^4^, ^5^ and possibly osteoblasts and chondrocytes.^57^ At the same time, FAP is a kind of skeletal muscle‐resident mesenchymal progenitors, which provides a supportive environment for myogenic cells by producing ECM components, such as collagens, laminin, and fibronectin.^17^ These proteins build a muscle stem cell niche and help them to renew.^58^, ^59^, ^60^

FAPs can directly produce cytokines and growth factors to regulate myogenesis and muscle maintenance, such as Bmp3b that activates Smad1/5/8 and the Akt pathway to participate in resting muscle homeostasis.^14^, ^15^, ^22^ The FAP depletion in muscle, which leading to the muscle weakness, muscle atrophy, and alterations in fiber type composition, ultimately leads to the failure of muscle mass maintenance.^22^ At the same time, FAPs play a role in maintaining NMJs.^22^ Genetic ablation of PDGFR‐α+ FAPs is facilitated by Cre mediated diphtheria toxin expression, which ultimately reduces the number of innervated NMJ.^55^ Specific studies have found that FAPs maintain the integrity of NMJ through its derived Bmp3b.^61^

Remarkably, the fate determination of FAPs has tight association with normal muscle growth and muscular pathological changes, and controlled by intracellular signaling pathways and local muscular environment. Changes in the local muscle microenvironment during muscle injury lead to rapid recruitment of eosinophils.^62^ At the same time, eosinophils secrete IL‐4 to promote FAP proliferation to support muscle regeneration while inhibit their differentiation into adipocytes.^18^, ^62^ Besides, IL‐15 signaling is activated, which stimulates the proliferation of FAPs by activating the Jak–STAT pathway.^63^ Subsequently, FAPs rapidly enter the cell cycle after injury and peak around 3–4 days after injury.^19^

On the one hand, activated FAPs contribute to muscle regeneration by promoting proliferation and differentiation of MuSCs in a paracrine manner. This process is mediated by several soluble molecules secreted by FAPs, such as follistatin (FST),^20^ WNT1‐induced signaling pathway protein 1 (WISP1),^21^ and bone morphogenetic protein 3B (BMP‐3B).^22^, ^64^ The active HGF presents in pharyngeal muscles was found to activate SC in mice and humans. At the same time, FAP is the main cell type that provides HGF, which can help the proliferation of SC in the pharyngeal muscles.^65^ Paradoxically, FAP‐derived IL‐6 has an apparent opposite effect in regeneration of injured muscles and atrophy of denervated muscles. Further research is needed to explain the different behaviors of FAP in different muscular environments.^66^ On the other hand, FAPs can also interact with other types of cells to aid muscle regeneration. FAPs serve as the main source of IL‐33 and highly expressed after muscle injury. IL‐33 can stimulate regulatory T cells (Treg) to proliferate around the injured area. Local expansion of Treg cells is an important condition for muscle repair.^66^, ^67^

Of course, the amount of FAPs is also precisely regulated by the muscle microenvironment. The number of FAP peaks about 96 h after muscle injury, then declines, and returns to pre‐injury levels over the next 5 days.^19^ Targeted clearance of FAPs is associated with infiltrating macrophages in the muscle. Specifically, apoptosis of FAPs is directly induced by TNF expressed by macrophages.^66^ FAP apoptosis induced by TNF signaling can inhibit fibroblast proliferation and pathological ECM deposition.^19^ After completing the muscle repair process, the muscle microenvironment returned to homeostasis again.

### 2.3 FAP in muscle microenvironment homeostasis disruption

Under the influence of multiple factors such as damaging factors (e.g. mechanical injury, oxidative stress, metabolic changes, and genetics) and aging, muscle microenvironment homeostasis is broken, which leads to the occurrence of pathological changes in muscles. For example, muscle atrophy (reduced repair capacity), fibrosis deposits, fat conversion and chronic inflammation. These pathological changes exacerbate the deterioration of muscle mass and function, which ultimately lead to the development of disease in the body.

#### 2.3.1 The link between FAP and muscle repair after homeostatic imbalance in the microenvironment

FAPs‐mediated reduction in muscle regeneration and repair is closely related to disruption of muscle microenvironment homeostasis, which is usually manifested by reduced regenerative function and abnormal signal communication between cells.

Increasing of age leads to changes in the stem cell population in the muscle microenvironment. The population of SCs in the resting muscle declines with age, resulting in insufficient muscle repair capacity.^68^ Numerous studies have shown that symmetric division of SCs due to aging leads to a decrease in quiescent satellite cell (QSC) populations (depletion of stem cell pools)^69^, ^70^ and ultimately to the decline in individual muscle mass,^71^ and NMJ degeneration.^72^ Of course, aging also affects FAPs, which ultimately contributes to the disturbance of the muscle microenvironment. Remarkably, FAP's support function for myogenesis is dysregulated by aging. WISP1 mRNA and protein expression are upregulated in young FAPs after muscle injury, but decreased secretion of WISP1 in aged FAPs leads to reduction in MuSCs function and impairment of muscle repair.^64^ Moreover, WISP1 is essential for efficient muscle regeneration which controls the expansion and asymmetric commitment of MuSCs via downstream Akt signaling. WISP1 secreted by FAPs promotes the generation of committed MuSCs by stimulating the asymmetric division of Pax7 + Myf5 cells. Studies suggest that the Akt pathway may be involved in the asymmetric self‐renewal of MuSCs. Systemic WISP1 treatment rejuvenates muscle source function and promoted muscle repair in elderly MUSCs.^21^

BMP3B is also known as growth differentiation factor 10 (GDF10), which is a member of the TGF‐β superfamily, and there is substantial evidence that BMP3B is also involved in the repair process of damaged muscles. The study found that BMP3B was specifically expressed by PDGFR‐α+ cells within skeletal muscle. However, Bmp3B expression is significantly reduced in aging muscle in mice and humans. And administration of recombinant BMP3B in older mice reversed their muscle‐reducing phenotype. In conclusion, BMP3B play an important role in myofiber mass maintenance but impaired in aged muscles.^22^

The intercellular molecular regulatory mechanism in which FAPs participate in muscle repair is significantly interrupted during aging. For instance, the IL‐33‐ST2 axis in FAP of aging mice decreased, which reduced the recruitment of Treg cells in skeletal muscle, and ultimately reduced the repair effect of damaged skeletal muscle.^67^

**FIGURE 1:**
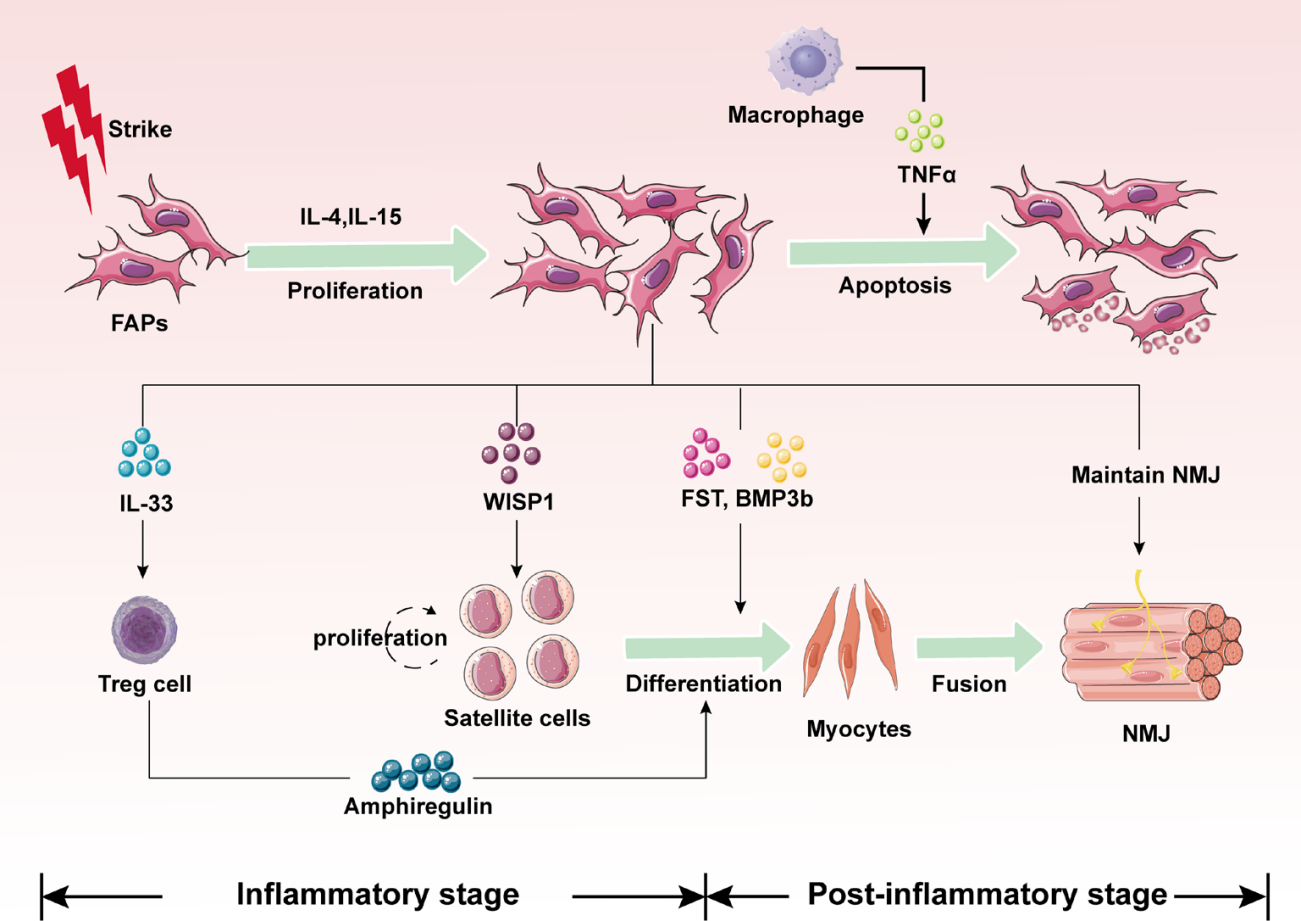
Muscles quickly enter the inflammatory phase after strikes, and inflammatory factors (IL‐4 and IL‐15) in the environment promote the rapid proliferation of fibro/adipogenic progenitors (FAPs) in a short period of time. FAPs play an important role in the muscle repair process. On the one hand, a large number of cytokines such as WISP1, FST, and BMP3b produced by FAPs can directly promote the proliferation and differentiation of satellite cells. On the other hand, FAPs can also secrete IL‐33 to act on Treg cells, which indirectly help the differentiation of satellite cells. In addition, the existence of FAPs is also important for the maintenance of neuromuscular junction (NMJ). However, the amount of FAPs do not keep growing, and as muscle repair is completed, FAPs are cleared by macrophages until it returns to normal levels.

Among hereditary muscle‐damaging disorders, DMD is one of the diseases of greatest concern. The disease is caused by mutations in the DMD gene encoding the protein dystrophin. Dystrophin deficiency causes muscle wasting, chronic inflammation which eventually progresses to muscle fibrosis, and fat accumulation.^73^, ^74^, ^75^, ^76^ The study found that muscle atrophy in DMD patients was directly related to decreased MuSC regeneration and improved after wild‐type MuSC transplantation.^77^ However, the effect of FAP on MuSC function in DMD patients remains unclear. It is well known that eosinophils help muscle regeneration after activating FAP through IL4 secretion during muscle homeostasis. Paradoxically, muscle eosinophilia was found in the muscles of mdx mice,^78^ which leads to collagen deposition and eventual death.^79^ Therefore, the mechanism of muscle repairing controlled by eosinophils and FAP in DMD deserves further study (Figure 1).

#### 2.3.2 Relationship between chronic inflammation accompanied by microenvironmental homeostatic imbalance and FAP

Inflammation caused by acute muscle injury helps muscle repair and regeneration. However, the imbalance of muscle microenvironment homeostasis caused by persistent inflammation may lead to the imbalance of inflammatory cells and abnormal behavior of FAPs.

**FIGURE 2:**
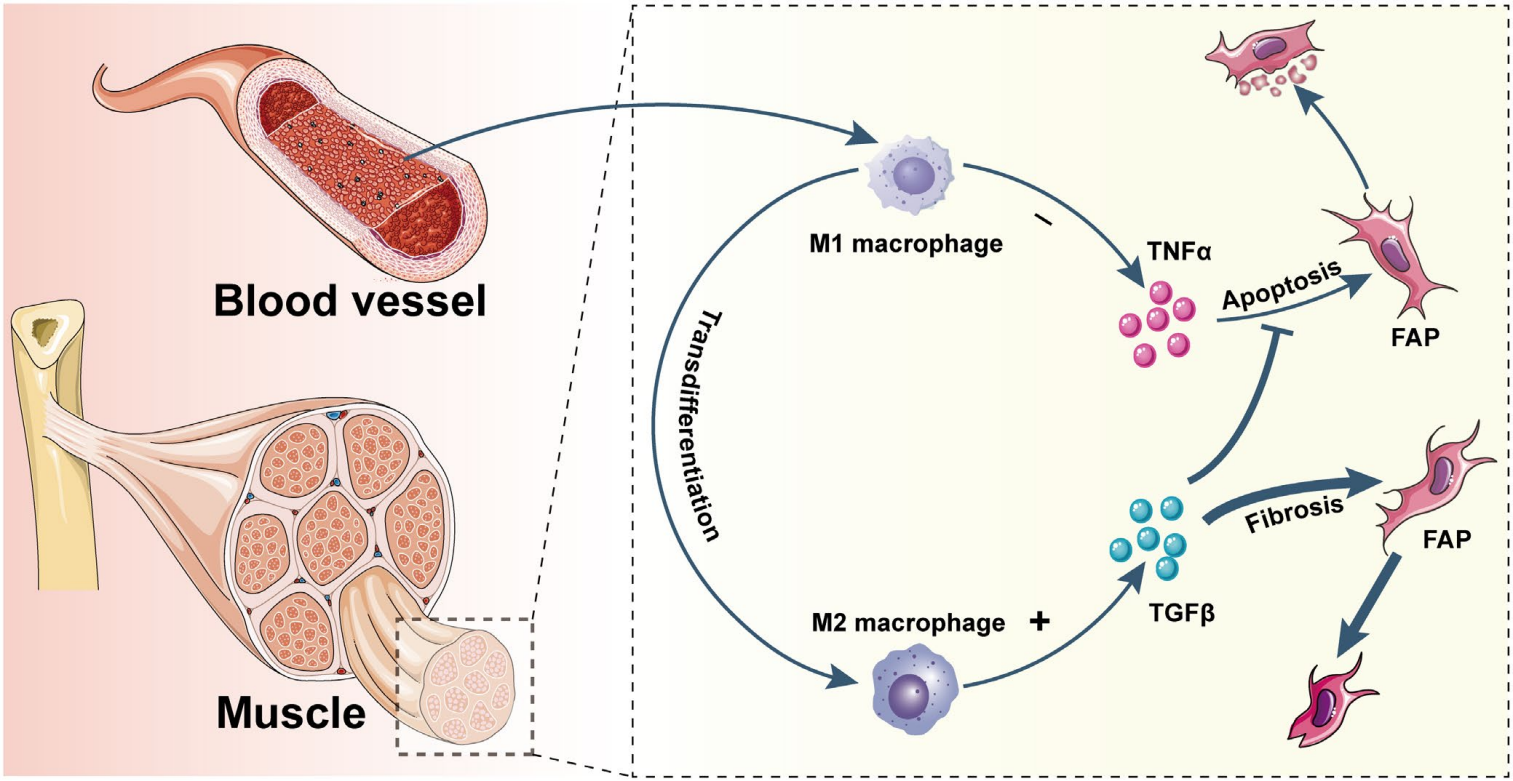
Inflammatory environment affects the fate of fibro/adipogenic progenitor (FAP). The damaged muscle tissue recruits large number of M1 macrophages under microenvironmental homeostasis. Macrophages secrete tumor necrosis factor (TNF)‐α to help clear excess FAPs in the muscle microenvironment. However, M2 macrophages transformed from M1 macrophages secrete large amount of TGF‐β to prevent the apoptosis of FAP and promote the fiber differentiation of FAP after the muscle microenvironment is broken. Therefore, the pro‐inflammatory and anti‐inflammatory balance of muscle microenvironment is closely related to FAP.

Under the condition of muscle microenvironment homeostasis, injured skeletal muscle can complete self‐repair and regeneration through normal inflammatory response. Early in muscle injury, neutrophils are responsible for recruiting M1 phenotypic macrophages and making them to produce inflammatory cytokines such as TNF‐α, IFN‐γ, and interleukin‐1β (IL‐1β).^80^, ^81^ And these cytokines help SCs proliferate.^82^, ^83^ After a rapid increase in M1 macrophages in the muscular environment, the transformation of M1 to M2 macrophages occurred. M2 macrophages produce cytokines that aid in the differentiation of SCs, such as IL‐4 and IL‐10.^52^ The behavior of FAPs is regulated by TNF‐α and transforming growth factor‐β1 (TGF‐β1), which are secreted by M1 and M2 macrophages, respectively. The balance of TNF‐α and TGF‐β1 during muscle microenvironment homeostasis contributes to FAP apoptosis in injured muscle and avoids abnormal deposition of ECM in skeletal muscle.^19^ However, disruption of the balance of these two cytokines in chronic inflammatory environment leads to excessive accumulation of ECM, resulting in poor muscle regeneration.^19^, ^66^ A persistent imbalance in the ratio of pro‐inflammatory and anti‐inflammatory macrophages in skeletal muscle leads to impaired activation and differentiation of SCs,^84^, ^85^ and increased cells and cytokines alter the composition of the ECM, leading to abnormal accumulation of fibrotic components^19^ (Figure 2).

#### 2.3.3 Association between FAP and muscle fibrosis deposition after microenvironmental homeostatic imbalance

Age‐induced muscle regulation imbalance and systemic changes are closely linked to the fibrous differentiation of FAP in muscles. The Notch signaling pathway is involved in the development of FAP‐driven fibrosis during aging. Notch signaling can maintain SC quiescence and promote proliferation after muscle injury, whereas insufficient upregulation of Notch ligand Delta leads to reduced notch activation in aging muscle, and the inhibition of Notch signaling leads to impaired propensity for resident precursor cells (SCs) to proliferate and produce myoblasts needed for muscle regeneration.^30^ Moreover, old myoblasts produce higher levels of TGF‐ß1 to inhibit differentiation, but Notch can restore the proliferation of senescent myoblasts by counteracting some of the antiproliferative effects of TGF‐ß.^86^

In the human heart, Notch activation leads to the increase number of cardiac precursor cells; however, the imbalance of Notch signaling causes abnormal development of fibrosis in heart. Thus, Notch signaling is a key mechanism that regulates the proliferation of mesenchymal cardiac progenitor cells and controls the balance between fibrosis development and cardiac repair in adults, but there is no more study on Notch signaling pathway in FAP.^87^

Numerous studies have shown that proteins secreted by immune cells also affect the regulation of FAP on muscle repair. Proteomics revealed that Metrnl is one of the secreted proteins of macrophages.^88^ Researchers found that Metrnl secretion decreased significantly after muscle injury in aging mice, leading to increased fibrosis in aging muscles. The authors describe the mechanism by which Metrnl treatment restores TNF‐α secretion in senescent macrophages, contributes to apoptosis of FAPs, inhibits profibrotic differentiation and fibrosis, and assists muscle regeneration in mice. In summary, the protein Metrnl secreted by macrophages tandem regulates FAP apoptosis and differentiation, which contributes to muscle regeneration and repair.^89^

Loss of SC‐dependent regenerative capacity does not exacerbate sarcopenia.^90^ However, it may lead to the increased fibrosis observed in older skeletal muscle. There is no further acceleration of sarcopenia in rodents during conditioned ablation of Pax7+ SCs. Although there is no direct effect on muscle fiber size, both ablation of Pax7+ SCs in muscle and a decrease in SCs due to aging increase levels of collagen deposition, which may result from fibrotic differentiation of FAP.^91^

In addition to muscle fibrosis caused by aging, hereditary myopathy can lead to muscle fibrosis. Transforming growth factor‐β is one of the most studied profibrotic factors.^92^ TGF‐β signaling is significantly upregulated in the skeletal muscle of DMD patients and mdx mice, and is involved in tissue fibrosis in multiple organs.^19^, ^92^, ^93^ Specifically, macrophages in dystrophic muscle secrete large amounts of TGF‐β1, while FAPs secrete larger amounts of enzymes, activating potential TGF‐β signaling.^94^ Overactivated TGF‐β signaling leads to increased fibrosis and decreased muscle regeneration.^94^, ^95^

Platelet‐derived growth factor receptor alpha (PDGFR‐α) is also involved in muscle regulation. In a physiological state, muscle development and angiogenesis are promoted in the embryo by activating PDGFR‐α signaling.^96^, ^97^ However, both excessive increase in PDGF ligands and enhanced PDGFR‐α activity lead to pathological fibrosis.^98^, ^99^ For example, the increased expression of PDGF‐AA in the muscles of patients with muscular dystrophy is accompanied by the activation of the downstream RhoA/ROCK2 pathway, which leading to the proliferation of FAPs and actin reorganization, and finally inducing muscle fibrosis.^100^, ^101^

Other studies have found that muscle denervation can also induce progressive accumulation of fibrosis.^102^ Muscle denervation leads to progressive accumulation of FAP, followed by activation of STAT3 in FAP and secretion of large amounts of IL‐6, which ultimately results in muscle atrophy and fibrotic changes. Abnormal activation of STAT3‐IL6 signaling in FAPs has also been found in some spinal cord injury diseases, such as amyotrophic lateral sclerosis (ALS) and spinal muscular atrophy (SMA).^102^

Similarly, muscle fibrosis can occur after irreversible mechanical trauma of muscles,^103^, ^104^ and the specific molecular mechanisms are still worth studying.

#### 2.3.4 Association between FAP and muscle fat infiltration after microenvironmental homeostatic imbalance

Xu et al. confirmed that glycerol‐induced muscle injury leads to high expression of intermuscular adipogenesis genes which is associated with the activation of FAP.^105^ On the one hand, the disruption of muscle microenvironment homeostasis caused by aging, oxidative stress, metabolic abnormalities, and irreversible muscle damage can lead to adipose differentiation of FAP. On the other hand, some non‐metabolic diseases and viral infections can also trigger muscle fat infiltration, but the specific regulatory mechanism is still not clear.^106^ A thorough understanding of the molecular mechanism between these external stimuli and muscle fat infiltration is of great significance for disease prevention and treatment.

Some immune cells are involved in the regulation of fat infiltration in aging muscle, such as inhibition of IL‐4 release by eosinophils promotes fat differentiation in FAP. In healthy mice body, eosinophils secrete IL‐4 to promote FAP proliferation to support muscle regeneration while inhibiting their differentiation into adipocytes.^18^ Specifically, IL‐4 in the muscle environment activates the STAT6 signaling pathway after binding to IL‐4R‐α on the surface of FAPs, stimulating the proliferation of FAP and strongly inhibiting the expression of fat genes. However, this signal pathway is interrupted by the elevation of glucocorticoids. Glucocorticoid levels are elevated in patients with cachexia caused by aging and chronic disease.^107^ Elevated glucocorticoid levels in mice inhibited IL‐4‐mediated signaling and ultimately increased lipid differentiation of FAP.^108^

Numerous studies have shown that Wnt signaling inhibits the differentiation of mesenchymal stem cells into adipocytes.^109^, ^110^, ^111^ WNT5a inhibits PPAR‐γ expression and FAP adipogenesis by activating β‐catenin signaling. However, increased level of active GSK3 in aging muscle led to decreased level of active β‐catenin. Eventually, changes in the WNT GSK3 β‐catenin axis led to increased expression of fat genes in FAP and increased muscle fat infiltration.^112^ Similarly, the downregulation of Wnt10b signaling promotes the expression of adipose genes in FAPs with age which leading to abnormal accumulation of intermuscular adipose tissue (IMAT).^113^ The Wnt signaling pathway is complex, and further research is needed to elucidate the mechanism of Wnt signaling and IMAT development during muscle damage and aging.

As a classical neurotransmitter, nitric oxide (NO) is also involved in the regulation of FAP fat differentiation, but this process is broken under conditions of muscle homeostasis imbalance. Neuronal nitric oxide synthase (nNOS) is gradually lost with muscle aging, which is the main source of NO in muscle.^114^ At the same time, the NO bioavailability of muscle tissue in the elderly is impaired due to increased oxidative stress.^115^ Studies have shown that NO can inhibit the differentiation of FAP into fat cells in vitro to regulate the progression of muscle diseases. Treatment with NO donor drugs significantly reduces the number of PDGFR‐α+ cells and alleviates the deposition of skeletal muscle fat and connective tissue. NO‐induced increased miR‐27b expression causes the suppression of peroxisome proliferator‐activated receptor gamma (Ppar‐γ1) expression, ultimately leading to inhibition of adipogenesis.^116^ These findings reveal another underlying mechanism by which FAPs impair muscle function with age, as well as therapeutic targets.

Muscle fat infiltration is not only affected by aging but is also strongly associated with age‐related metabolic diseases such as reduced insulin sensitivity and type 2 diabetes (T2D). Skeletal muscle is an important part of the body that processes glucose, and muscle glucose utilization is closely related to insulin sensitivity, which is crucial for the development of systemic insulin resistance and hyperglycemia.^117^ Muscle mass, muscle strength, and function getting worse with age in T2D patients, while the increase in ectopic lipids in cells and skeletal muscle interstitial are associated with reduced insulin sensitivity.^24^, ^25^, ^26^ Previous literatures have shown that mouse muscle‐derived stem cells transdifferentiate into adipocytes when exposed to high glucose environments in vitro, suggesting a potential link between fat cell accumulation and the systemic environment.^118^, ^119^ Evidence from subsequent studies suggests that IMAT accumulation in skeletal muscle is closely related to mesenchymal stem cell FAPs.^120^, ^121^ In addition, FAP‐derived adipocytes have lower insulin sensitivity compared with conventional adipocytes, suggesting that excessive accumulation of FAP‐derived adipocytes may lead to impaired peripheral insulin sensitivity.^6^

Retinoic acid signaling is also involved in the regulation of muscle fat infiltration.^122^ Retinoic acid signaling helps maintain the undifferentiated state of FAPs and promotes their proliferation in the early stages of muscle regeneration. Subsequent studies found that decreased RA signaling in skeletal muscle of obese mice leads to fatty infiltration and fibrosis in skeletal muscle.^28^ Fat and fibrotic differentiation of FAPs can be inhibited by RA supplementation, which contributes to the regeneration of obesity‐damaged muscles.^123^ Therefore, RA signaling in FAP can serve as a new strategy to inhibit muscle loss and improve muscle function.

In young mdx mice, FAP is not sensitive to the anti‐fat effect of NOTCH signaling, but does not cause muscle fat infiltration due to the inflammatory environment.^124^ Macrophage infiltration in the muscle of aged mdx mice is significantly reduced with age, and the insensitivity of NOTCH signaling makes lipogenesis inhibition insufficient to prevent fat deposition.^125^ Enhanced NOTCH signaling reduces adipose differentiation of FAP and adipose tissue infiltration both in vitro and in vivo.^61^, ^126^

**FIGURE 3:**
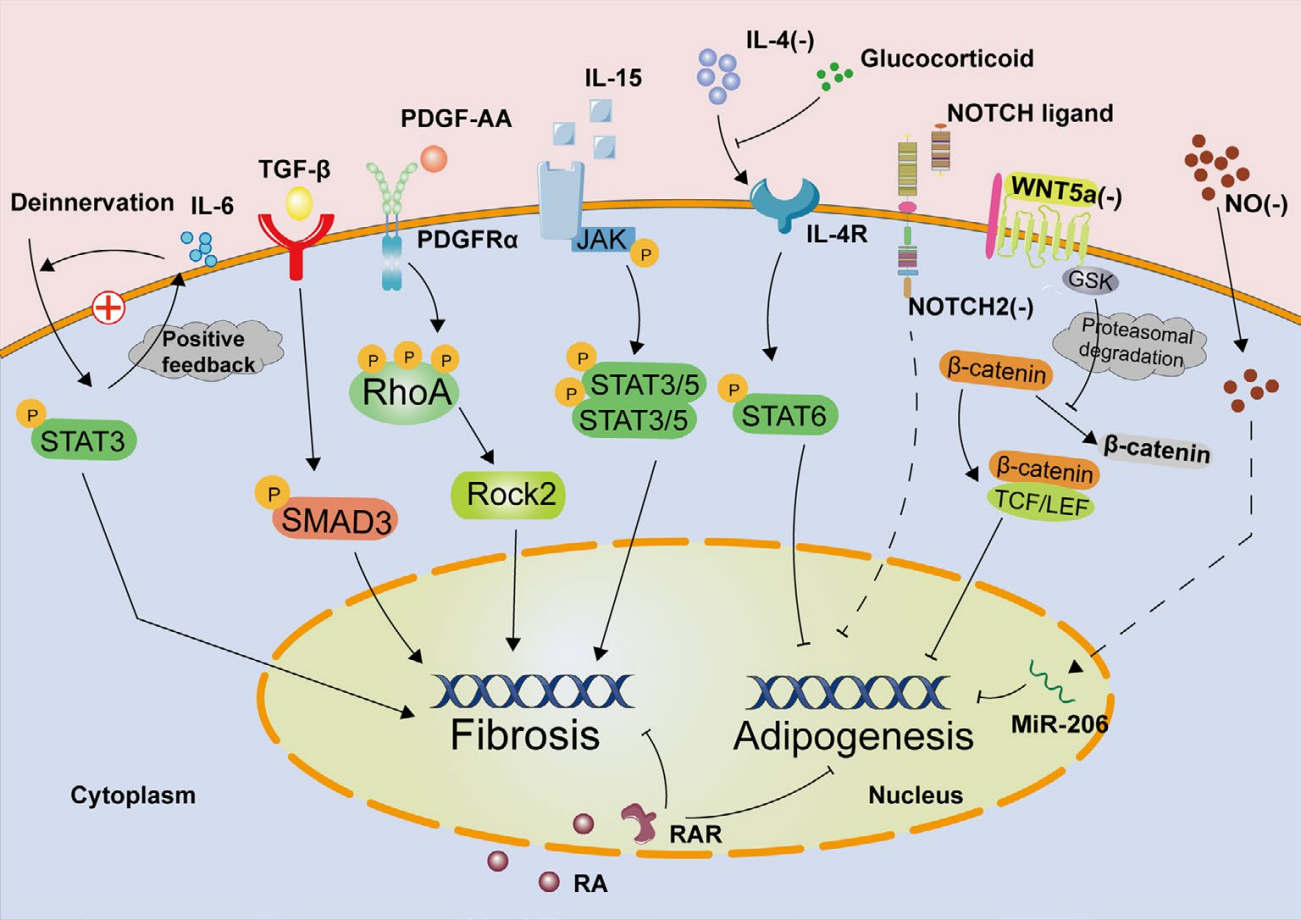
After muscle microenvironment homeostasis is broken, the signal network composed of inflammatory factors, cytokines, and cell membrane ligands was disrupted, resulting in increased transcription of fibrotic genes and decreased inhibition of fat transcription signals in fibro/adipogenic progenitor (FAP) nuclei. Abnormal differentiation of FAP eventually causes pathological changes in muscles.

The irreversible muscle lesions caused by mechanical muscle injury are also closely related to FAPs. FAPs participate in muscle regeneration through transient proliferation after acute skeletal muscle injury and provide an appropriate environment for muscle fiber repair by activated muscle SCs and downstream myogenic progenitor cells (MPs).^4^, ^19^ After completing the regeneration procedure, FAPs initiate the apoptosis procedure to restore their numbers to baseline levels. Conversely, FAPs apoptosis signaling is disrupted in chronic muscle injury which leading to failure of muscle repair.^19^ Rotator cuff tear is a common tendon injury.^127^ The onset of rotator cuff disease begins with a primary rotator cuff tendon tear, and secondary damage occurs as the disease progresses.^128^ FAPs have been identified as the primary source of muscle fat infiltration after rotator cuff tears.^103^ However, the specific mechanism of muscle degeneration caused by mechanical muscle injury remains unclear. What's more, the amount of interstitial fat infiltration is directly related to decreased muscle function and injury severity^129^ (Figure 3).

## 3 THE ROLE OF FAP AS CELL TARGETS IN THE TREATMENT OF MUSCLE‐DAMAGING DISEASES

Aging and chronic inflammation lead to the abnormal behavior of FAPs after the imbalance of muscle microenvironment homeostasis. Specifically, persistent inflammation of the muscle caused by damaging factors leads to abnormal function of FAPs, resulting in decreased muscle repair and exacerbating fibrotic deposition and fat infiltration. The combinations of various pathological changes constitute the clinical presentation of a particular disease. These disorders include sarcopenia, DMD, rotator cuff tear, IVDD (disk degeneration), and muscle degeneration due to T2DM, which ultimately leads to impaired daily activities and physical disability. According to the characteristics of these muscle‐damaging diseases, we identify key cells that affect the pathological changes in damaged muscle tissue, FAPs, and by targeting FAP to control disease progression and improve prognosis and daily functioning of patients.

### 3.1 Sarcopenia

The manifestation in reduced muscle mass and strength are called sarcopenia.^130^ Sarcopenia is classified as a disease state and was granted the clinical code ICD‐10‐CM (M62.84) in 2016.^131^ The decline in skeletal muscle mass and muscle function caused by sarcopenia leads to reduced physical activity, disability, and increased mortality in older adults.^132^, ^133^ Depending on the etiology, sarcopenia can be divided into primary and secondary sarcopenia. Primary sarcopenia refers to the loss of muscle mass and function induced by aging, while secondary sarcopenia refers to the loss of muscle mass caused by specific diseases, such as malignant tumors and chronic obstructive pulmonary disease.^134^ The incidence of both types of sarcopenias increases with age. The diagnosis of sarcopenia is mainly accomplished by assessing muscle mass and measuring the physiological performance of the muscles.^135^ There are many causes of sarcopenia, such as NMJ dysfunction, imbalances of muscle growth factors and inhibitors, variable declines in several hormones, chronic inflammation, and worsening lifestyle and nutritional status.^136^ Chronic inflammation is prevalent in the elderly population, which plays a key role in the occurrence and progression of sarcopenia, such as the imbalance of anti‐inflammatory and pro‐inflammatory cytokines.^137^, ^138^ Another important feature of sarcopenia is the loss of lean muscle mass, which is primarily due to the reduction in muscle fibers, especially type II fibers.^139^ Decreased amount of type II fiber is also associated with decreased muscle strength in sarcopenia.^140^ In addition, sarcopenia is often accompanied by the reduction in SCs and motor units, which are an important reason for the occurrence of sarcopenia.^141^, ^142^

Sarcopenia should be treated with nonpharmacologic and pharmacological approaches based on the mechanism of pathophysiological changes. Non‐pharmacological interventions for sarcopenia include resistance exercise and appropriate nutrition. On the one hand, lack of physical activity is associated with loss of muscle mass, so physical exercise is considered the cornerstone of the treatment of sarcopenia.^143^ Specifically, resistance training (RT) of muscles increases hormone concentration and protein synthesis rate, which can effectively prevent and treat sarcopenia.^144^ On the other hand, nutritional supplements include vitamin D and branched‐chain amino acids, both of which can significantly increase muscle mass and strength for body function. Therefore, resistance exercise combined with nutrition can significantly enhance muscle strength and function, ultimately improving the prognosis of sarcopenia.^145^, ^146^ So far, there is no specific drug approved by FDA to treat sarcopenia. Recommended treatment is approved with drugs, such as growth hormone, anabolic steroids, selective androgen receptor modulators, myostatin inhibitors, II receptor‐activating drugs, beta‐blockers, and ACE inhibitors. But the effectiveness of these drugs varies among different populations. Growth hormone increases muscle protein synthesis and increases muscle mass, but does not improve muscle strength or function.^147^ Testosterone or other anabolic steroids have also been studied. These drugs have some positive effects on improving muscle mass and strength, but their use is limited due to adverse drug reactions, such as increased risk of prostate cancer in men and increased risk of cardiovascular events in women.^147^, ^148^ Bimagrumab is a monoclonal antibody against the activin II receptor that increases lean muscle mass and functional status.^149^ Beta receptor blockers, ACE inhibitors, and troponin activators have positive effects on muscle mass and grip strength.^149^ Senescent cells in tissues can release SASP factors, including exosomes, and have profound impacts on the growth and function of normal cells. For example, SASPs (including extracellular vesicles [EVs]) inhibit the normal function of muscle progenitor/stem cells (MPCs). Removal of senescent FAPs with drugs such as senolytics can effectively rescue the number and function of MPCs and repair muscle damage in mice with muscular dystrophy.^150^ Some cytokines can also be used to treat sarcopenia, such as Metrnl. Studies have shown that Meteorin‐like (Metrnl) gets involved in various regulation of cells and is closely related to adipose tissue metabolism and muscle regeneration in the body.^151^, ^152^ Furthermore, peptide injection of recombinant Metrnl counteracts the profibrotic process by TNF‐induced apoptosis of FAP cells. This demonstrates the therapeutic application of METRNL in improving aging muscle.^89^

### 3.2 Duchenne muscular dystrophy

Duchenne muscular dystrophy is a pretty severe progressive atrophic muscle disease caused by a mutation of the DMD gene on the X chromosome, resulting in the loss of functional muscular dystrophy protein.^153^ Current estimates of DMD from global studies indicate that the incidence of DMD is about 1:5000.^154^ Dystrophin is a cytoskeletal protein that connects the cytoskeleton to the ECM through its amino terminal and carboxyl terminal domains, respectively, thus acting as an important stabilizer of muscle fibers during locomotion.^155^, ^156^ Due to the lack of dystrophin, muscle fibers are damaged during contraction and cause muscle inflammation that inhibits muscle fiber regeneration. Eventually, these alterations lead to fibrosis and adipositization of the muscles.^157^, ^158^ In patients with DMD, serum creatine kinase values are elevated from birth, which may be helpful for early neonatal diagnostic detection.^159^ Over time, muscle fibers degenerate, and the fat and connective tissue between muscle fibers increase,^160^ changes in the size of muscle fibers and inflammatory cell infiltration.^161^ Glucocorticoids, as a conventional treatment for DMD, can delay symptoms by reducing inflammation and lymphocyte reaction.^162^ Studies have shown that the expression of PDGF‐AA is higher in the skeletal muscle of DMD patients,^163^ and PDGF‐AA induces RhoA ROCK2 pathway signaling in DMD‐FAP and helps FAP proliferation, ultimately leading to muscle fibrosis. With a well‐known ROCK inhibitor, Fasudil, the effect of PDGF‐AA on cells is blocked in vitro, which reduces muscle fibrosis and restore muscle strength in a mouse model of DMD.^101^ Therefore, ROCK2 signaling inhibition may be a new therapy for the treatment of DMD. On the contrary, epigenetic drug modifiers such as HDACi have therapeutic effects on dystrophic muscles.^164^ HDACi inhibits the adipogenic potential of dystrophic FAPs while enhances the ability of FAPs to secrete follistatin to support the myogenic differentiation of MuSCs.^20^ Ultimately, it promotes endogenous muscle regeneration and inhibits fibro–adipogenic degeneration of muscle.^165^ FAPs in the muscles of DMD patients or mdx mice exposed to HDACi elevates miR‐206 levels in EVs, which reduce muscle fibrosis and enhance regeneration.^166^ Notably, miR‐206 also inhibits the lipogenic differentiation of FAPs by targeting Runx1 transcripts.^167^ Therefore, HDACi is promising for the treatment of DMD patients. Other studies have shown that drug consumption of mesenchymal cells in mouse models of DMD can ameliorate muscle damage and delay loss of muscle function. In mouse models of DMD, genetic ablation of FAPs induced by the suicidal transgene, viral thymidine kinase (TK), can reduce muscle damage and preserve muscle function.^168^ This may be a new direction for the treatment of DMD.

Imatinib is a drug that inhibits regulators of tyrosine kinase activation. Studies have shown that imatinib can inhibit PDGF‐AA that induced the proliferation of muscle mesenchymal progenitor cells and their fibrotic gene expression, ultimately alleviating muscle fibrosis in mdx mice.^169^ Therefore, imatinib as a widely used drug also plays an important role in the treatment of muscle fibrosis.

Studies have shown that long‐term treatment with the NO donor drug, molsidomine, reduces the amount of FAP in skeletal muscle tissue and inhibits adipose tissue deposition and indirect fibrosis in mdx mice.^116^ The NO produced by the drug can directly affect the regulatory function of miR‐133a^170^ (a regulator of collagen 1A1 expression) and miR‐27b^171^ (a key inhibitor of peroxisome proliferator‐activated receptor gamma [*Ppar‐γ*]), ultimately aiding the treatment of DMD.^116^

### 3.3 Rotator cuff tears

Rotator cuff disease is one of the most common cause of shoulder disability and is particularly prevalent in older people, where rotator cuff tears account for a large proportion. There are two main causes of rotator cuff tears: injury and degeneration. The injury usually presents as an acute tear of the rotator cuff. This type of tear can occur alone or be accompanied by other shoulder injuries, such as a broken collarbone or a dislocated shoulder.^172^ Usually, rotator cuff tears are common in the dominant arm.^172^ However, degenerative changes after tearing are more common and closely related to age. The incidence of rotator cuff tears increases with age, with 20% and 80% of patients in their 60s and 80s, respectively.^173^

Studies have demonstrated that tendon retraction caused by rotator cuff tendon rupture can cause muscle fat infiltration and muscle atrophy.^174^ Moreover, Muscle fat infiltration and atrophy further affect tendon healing.^175^ Fatty degeneration is also an independent risk factor for recurrent rotator cuff tears.^176^ Regarding the treatment of rotator cuff tears, a small molecule inhibitor, CWHM‐12, has been shown to reduce FAP‐derived fibrosis in vitro.^177^ In addition, the literature shows that SB431542 can promote apoptosis of FAP by inhibiting TGF‐β1 signaling, ultimately leading to significant improvements in fibrosis, fat infiltration, and muscle weight loss.^178^ Unlike classical adipocytes, adipocytes differentiated by FAP have a unique signature expressing uncoupling protein 1(UCP‐1), which is the hallmark of beige adipose tissue (BAT).^179^, ^180^ Coincidentally, the fat that accumulates in the muscle after RC tear has BAT characteristics. And impaired beige fat activity led to more severe RC muscle atrophy and FI. The study found that there is a selective β3‐adrenergic receptor agonist called Amibegron that can prevent and reverse RC atrophy and fat infiltration by promoting BAT activity.^181^ It is a new way to treat damaged muscles.

### 3.4 Intervertebral disc degeneration

Low back pain (LBP) is a very common condition with a prevalence of 20.3% in the adult population. And the prevalence is increasing rapidly in people over 30 years of age.^182^ More than 80% of adults experienced at least one chronic LBP in their lifetime, usually associated with lumbar disk herniation.^183^ With the progression of lumbar disease, paravertebral muscle changes occur, including muscle atrophy, increased fat and connective tissue in the muscle, changes in the type of muscle fibers, changes in the muscle interstitial cell population, and altered gene expression.^184^, ^185^, ^186^, ^187^ These changes may be related to denervation and reinnervation of paravertebral muscles due to disk herniation and nerve root compression.^188^ It also has been suggested that greater fat infiltration is associated with inflammatory dysregulation found in multifidus muscle in patients with degenerative spine.^189^ At the microscopic scale, histological analysis of animal and human paravertebral muscle biopsies also confirmed the outcome of lumbar degeneration leading to muscle fibrosis, inflammation, and vascularization.^190^ Of course, muscle fat infiltration increases with age, age‐dependent fatty degeneration occurs earlier and more severely in the erector spinae than in the multifidus muscle, and the trend of increased paraspinal fat infiltration is more pronounced in the affected women.^191^ As the disease progresses, the proportion of IIB (fast muscle glycolytic) fiber in the muscle of low back pain patients is significantly higher than that of type I (slow oxidation) fiber.^186^

The multifidus muscle tissue of patients with lumbar disk herniation contain high levels of adipose infiltration and fibrosis, as well as elevated expression of fibrosis and adipose genes. This is related to the large number of FAP and SC in the multifidus muscle of patients with disk herniation.^187^ It has a stronger tendency to fat infiltration and fibrosis after the multifidus muscle is disturbed. However, the treatment of paravertebral muscle degeneration has not received enough attention, and subsequent treatments are still being explored.

### 3.5 Muscle degeneration due to T2DM

Type 2 diabetes mellitus (DM) is a complex chronic systemic disease with metabolic disorders, including hyperglycemia, hypertriglyceridemia, and insulin resistance.^192^ With the aging of the population and the extension of life expectancy, the number of people with T2D is increasing dramatically in the world, especially with obesity, decreased levels of skeletal muscle contraction and physical activity. Between 2017 and 2045, the global population of adults aged 65 and older with diabetes is expected to increase from 122 million to 253 million.^193^ The main tissue affected by disrupted glucose metabolism is skeletal muscle, and defects in metabolic signaling in muscle tissue can lead to systemic insulin resistance.^194^ Intermuscular adipose tissue (IMAT) is defined as the visible fatty tissue muscle mass below and between the muscle fascia. It is inversely correlated with insulin sensitivity in patients with T2D.^195^ In addition, ECM deposition in skeletal muscle of obese mice is also associated with insulin resistance.^196^, ^197^ Thrombospondin 1 (THBS1) is an adherent ECM glycoprotein expressed primarily in visceral adipose tissue, and its expression is elevated in insulin‐resistant individuals.^198^, ^199^ Thrombospondin 1(THBS1) activates transforming growth factor beta (TGF‐β) signaling to promote mesenchymal cell proliferation, leading to fibrosis of muscle in obese mice.^196^, ^200^, ^201^ A variety of factors such as intermuscular adipose tissue (IMAT) and muscle fibrosis contribute to progression and muscle weakness in people with T2D. The mechanisms of muscle fat deposition and fibrosis caused by T2D are unknown and require further study. As a conventional treatment for T2D, metformin is effective in blocking muscle interstitial fibrosis and fat formation. Specifically, metformin treatment reduces the number of FAPs in T2DM patients and impairs their ability to differentiate into fats and fibers^202^ (Table 2).

**TABLE 2:** Pathological changes and therapeutic measures of muscle‐damaging diseases.

Diseases	Etiology	Mechanisms	Pharmacological interventions	References
Sarcopenia	Aging, dysfunction of neuromuscular junctions, inflammation, hormones	Decreased muscle fibers	Bimagrumab	[130, 140]
		Satellite depletion	Senolytics	[132, 133, 141]
		Fatty infiltration, fibrosis	Metrnl	[83, 142, 143]
DMD	Genetic mutation	Fibrosis	Fasudil, HDACis, imatinib, molsidomine	[94, 148, 151, 155, 157, 160]
		Fatty infiltration	HDACis, molsidomine	[20, 148, 151, 161]
		Inflammation	Glucocorticoids	[148, 152, 153]
Rotator cuff tear	Aging, mechanical injuries	Fibrosis	CWHM‐12	[169]
		FAP apoptosis inhibition	SB431542	[116]
		Muscle atrophy, fat infiltration	Amibegron	[166, 172]
IVDD	Aging, lack of exercise	Denervation, muscle atrophy, fat infiltration	Unclear	[175, 176, 177, 178, 179]
Type 2 diabetes	Aging, endocrine disorders, obesity	Metabolic disorders, insulin resistance, fat infiltration and fibrosis	Metformin	[185, 186, 187, 188, 189, 190, 191, 192, 193]

Abbreviations: FAB, fibro/adipogenic progenitors; HDACis, histone deacetylase inhibitors; IVDD, intervertebral disk degeneration; SC, satellite cells.

## 4 CONCLUSION AND PERSPECTIVE

As a common manifestation of muscle degeneration, muscle weakness is often associated with factors such as aging and irreversible damage of muscles which ultimately leads to impaired mobility and function. Numerous studies have confirmed that the deterioration of the muscle microenvironment is closely related to muscle weakness. In order to investigate the main factors affecting muscle microenvironment homeostasis, we selected a kind of mesenchymal stem cell that can specifically express PDGFR‐α cell surface markers as the study object. This article discusses the construction of muscle microenvironment, FAP's participation in muscle microenvironment homeostasis, the pathological mechanism of muscle homeostasis disruption, the characteristics of specific muscle diseases and their corresponding therapeutic targets. As a kind of pluripotent progenitor cell, FAPs can differentiate into fibroblasts, adipocytes, osteoblasts, and chondrocytes under suitable conditions. Although myogenic differentiation does not occur, FAPs can generate ECM to build a suitable extracellular environment for muscle regeneration, express a variety of cell growth factors and interact with other types of cells to help muscles repair, maintain the number of NMJs to support muscle strength. FAPs play an important role in the construction and maintenance of muscle microenvironment homeostasis. However, under the influence of age and injury factors, the homeostasis of muscle microenvironment out of balance which causes the abnormal signal of FAPs, eventually leads to the abnormal accumulation and differentiation of FAPs. Pathological changes in muscles such as muscle atrophy, fibrosis, fat infiltration, and chronic inflammation occur after long‐term imbalance. Although the types of diseases that cause muscle weakness are different, the pathological changes in the muscles are basically similar, so the occurrence and progression of muscle lesions can be reversed by targeting the abnormal signaling pathway of FAPs.

The fate of FAPs is largely influenced by the muscular environment. PDGFR‐α+ cells isolated from glycerol‐injected muscle transplanted into CTX‐injected muscle did not differentiate into adipocytes. However, PDGFR‐α+ cells isolated from CTX‐injected muscles differentiated into C/EBP‐α+PPAR‐γ+ adipocytes in glycerol‐injected muscles.^5^ These results indirectly suggest that improving the muscle microenvironment plays a very important role in controlling the progression of muscle pathology. Therefore, improving the damaged muscle microenvironment is more important than controlling the number of FAPs and the direction of differentiation, which may be a new idea for the treatment of muscle diseases in the future. There are still many questions worth investigating, such as the mechanism of FAPs and SC on the maintenance of NMJ, the relationship between other types of muscle diseases and FAPs, the effect of exercise on damaged muscles, and epigenetic regulation.

In addition to the regulation of muscle homeostasis, the studies of FAPs in osteogenic differentiation and cartilage differentiation in vivo are also of great significance. Moreover, in spinal degenerative diseases, the occurrence of paravertebral muscle degeneration is accompanied by the process of disease progression, and the paravertebral muscle degeneration further aggravates the body's dysfunction. Studies have found that FAPs are involved in the degeneration of paravertebral muscle, but the in‐depth mechanism and targeted therapy need more discussion.

## AUTHOR CONTRIBUTIONS

Kai Yin, Chengmin Zhang, Chuan Yang, Xiaoyu Wei, Zihan Deng, Can Chen, and Yueqi Chen collected and reviewed the literatures. Kai Yin, Chengmin Zhang, Chuan Yang, Zihan Deng, Can Chen, Yueqi Chen, and Fei Luo drafted and revised the manuscript. Kai Yin, Tingwen Xiang, and Yueqi Chen have drawn the pictures and tables. Yueqi Chen, and Fei Luo designed and conceived the review. All authors read and approved the final manuscript.

## DISCLOSURES

The authors declare that they have no known competing financial interests or personal relationships that could have appeared to influence the work reported in this paper.

## Data Availability

All data are available from the corresponding authors upon reasonable request.
